# Atualizações em enxertos e substitutos ósseos

**DOI:** 10.1055/s-0045-1814115

**Published:** 2025-12-30

**Authors:** Edgard Eduard Engel, Nelson Fabrício Gava, Mariana Avelino dos Santos, Leonardo Gomes Baldoino, Lucas Klarosk Ismael, Luis Felipe Miras Modolo

**Affiliations:** 1 Departamento de Ortopedia e Traumatologia, Hospital das Clínicas, Faculdade de Medicina de Ribeirão Preto, Universidade de São Paulo, Ribeirão Preto, SP, Brasil

**Keywords:** cimentos ósseos, regeneração óssea, remodelação óssea, substitutos ósseos, bone cements, bone regeneration, bone remodeling, bone substitutes

## Abstract

Os defeitos ósseos, causados por traumatismos, infecções, neoplasias e outras condições, são comumente tratados com enxerto autólogo, que é considerado o padrão-ouro devido às suas propriedades de osteoindução, osteocondução e osteogênese. No entanto, seu uso apresenta limitações, como a disponibilidade restrita, a morbidade no local doador e o aumento do tempo cirúrgico. Como alternativas, enxertos alógenos, xenógenos e substitutos ósseos sintéticos, que incluem cerâmicas, biovidros, resinas e metais, têm sido desenvolvidos, sendo frequentemente modificados com o acréscimo de elementos osteoindutores, como fatores de crescimento e íons bioinorgânicos. O substituto ósseo ideal deve ser biocompatível, bioabsorvível, mecanicamente resistente, poroso e capaz de promover osteointegração. Embora os substitutos sintéticos tenham avançado, ainda não alcançaram a eficácia do enxerto autólogo, principalmente no que se refere à osteointegração e à viabilidade econômica. Contudo, as inovações em biologia molecular, proteínas ósseas e terapias genéticas oferecem perspectivas promissoras para o desenvolvimento de novos biomateriais. Este artigo pretende apresentar ao leitor o tema dos substitutos ósseos, com uma classificação dos grupos de materiais utilizados e as principais características de cada grupo.

## Abstract (en)

Bone defects caused by trauma infections neoplasms and other conditions are commonly treated with autologous grafts which are considered the gold standard due to their osteoinduction osteoconduction and osteogenesis properties. However their use has limitations including limited availability donor-site morbidity and increased surgical time. Alternatively allogeneic grafts xenografts and synthetic bone substitutes including ceramics bioglasses resins and metals have been developed and are often modified with osteoinductive elements such as growth factors and bioinorganic ions. The ideal bone replacement should be biocompatible bioabsorbable mechanically resistant porous and capable of promoting osseointegration. Although synthetic substitutes have advanced they have not yet achieved the effectiveness of autologous grafts especially concerning osteointegration and economic viability. However innovations in molecular biology bone proteins and gene therapies offer promising prospects for the development of new biomaterials. The current article introduces the reader to bone substitutes by presenting a classification of the materials used and the main characteristics of each group.

## Introdução

Defeitos ósseos são uma condição clínica enfrentada por ortopedistas, neurocirurgiões, cirurgiões de cabeça e pescoço e odontologistas. Na prática ortopédica, esses defeitos podem resultar de traumatismos de alta energia e de grandes ressecções ósseas por diferentes patologias, tais como tumores, infecções, soltura de próteses ou complicações na consolidação óssea. O preenchimento do defeito ósseo tem como objetivo restaurar a estrutura do osso e, principalmente, a resistência mecânica. 1 2 As alternativas disponíveis para esse preenchimento podem ser agrupadas em enxertos (materiais biológicos) e substitutos (materiais sintéticos). 2

O enxerto ósseo esponjoso autólogo é reconhecido como o padrão-ouro para a correção de defeitos ósseos ou incremento de tecido ósseo ( *augmentation* ). No mundo, a cada ano são realizadas mais de dois milhões de cirurgias ortopédicas nas quais se colhe enxerto ósseo, que corresponde ao segundo tipo de transplante mais realizado, atrás apenas da transfusão sanguínea. 2 O osso autólogo é considerado o enxerto ideal, pois é completamente compatível e tem todas as características indispensáveis para a osteointegração: osteoindução, osteocondução e osteogênese. 1 3 No entanto, a disponibilidade limitada do enxerto, o potencial morbidade do sítio doador e o aumento do tempo da cirurgia impõem a busca de alternativas. 2 4

No caso dos defeitos ósseos, vários tipos de biomateriais estão sendo criados e testados, em uma colaboração de áreas como a medicina, a biologia, a química, a engenharia e a odontologia. 4 5

Os objetivos deste artigo são apresentar as alternativas de tratamento dos defeitos ósseos e sua classificação, e discorrer sobre os princípios teóricos da integração dos substitutos ósseos e as tendências futuras no desenvolvimento de novos biomateriais.

## Definições

De forma ampla, os enxertos podem ser considerados substitutos ósseos, pois preenchem os critérios de biomateriais. No entanto, esse termo é mais frequentemente utilizado para descrever materiais sintéticos que são produzidos a partir de elementos encontrados na natureza e podem ser classificados em cerâmicas, polímeros, biovidros e metais. 6 *Biomateriais* são, portanto, materiais naturais ou sintéticos destinados a replicar a forma e/ou a função de diferentes tecidos humanos ou de animais, e podem atuar de forma permanente ou transitória. 7 Os substitutos ósseos podem ser utilizados para o preenchimento de defeitos, a substituição de segmentos ósseos, e o reforço e a estimulação do processo de consolidação óssea em diversas regiões do corpo de forma percutânea ou aberta. Devem ser estruturalmente semelhantes ao osso, mecanicamente resistentes, fáceis de manusear, seguros e ter custo acessível. 8

Algumas características dos substitutos ósseos são extremamente importantes. Eles devem apresentar *biocompatibilidade* ou, pelo menos, biotolerância, para garantir a assimilação adequada aos tecidos biológicos. *Biocompatibilidade* significa que o organismo aceita o material naturalmente, e interage com ele do ponto de vista metabólico, ao passo que a biotolerância indica que ele é aceito pelo organismo sem causar reações adversas significativas, mesmo que não favoreça uma resposta ideal. 3

A *osteointegração* é a principal propriedade esperada de um substituto ósseo, e caracteriza-se como o processo de interdigitação entre o tecido ósseo do hospedeiro e o implante. Nesse processo, o osso receptor gera tecido ósseo novo que penetra nas irregularidades microscópicas da superfície do substituto, o que garante a sua estabilização e a sua fixação na região de inserção. 1 8 Essa integração biológica e mecânica é crucial para a funcionalidade e a longevidade do implante. 2 6

A osteointegração depende dos seguintes processos: osteogênese, osteocondução e osteoindução. *Osteogênese* é a capacidade de o substituto promover a formação de novo tecido ósseo; 3 na prática, somente o enxerto autólogo apresenta tal propriedade. *Osteocondução* é a propriedade estrutural do material que serve como suporte físico para o crescimento e a regeneração do tecido ósseo. O material osteocondutor guia e facilita a invasão e o crescimento de células ósseas, vasos sanguíneos e matriz óssea nos locais onde a regeneração óssea está ocorrendo. 3 Tal propriedade está intimamente relacionada às características da superfície do material, bem como às dimensões de seus poros, canais ou poços. *Osteoindução* refere-se à capacidade de biomaterial estimular células indiferenciadas a se diferenciar em osteoblastos, que são as células responsáveis pela formação do tecido ósseo, 3 o que promove a osteogênese.

**Fig. 1:**
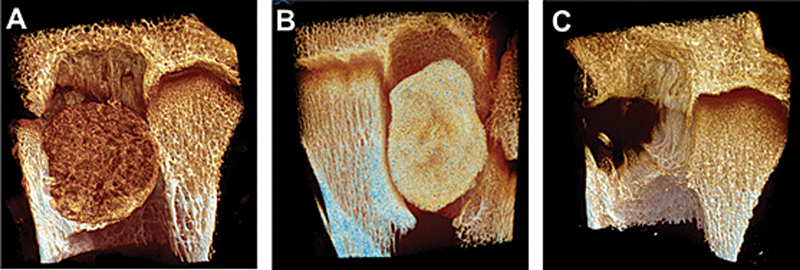
Comparação entre falhas ósseas preenchidas com substituto ósseo poroso ( **A** ), bloco sólido de cimento ( **B** ) e sem preenchimento ( **C** ), 3 meses após a cirurgia em carneiros, analisadas por microtomografia. Em ( **A** ), o contato entre o osso e o substituto é íntimo, o que indica a osteointegração. Em ( **B** ), ocorre a reabsorção do osso receptor em volta do bloco de cimento e a formação de tecido fibroso. No defeito não preenchido ( **C** ), observa-se a formação de espículas ósseas recém-formadas procurando invadir o defeito.

Em relação à estrutura do substituto ósseo, é recomendável que sua morfologia seja análoga à do osso a ser substituído, seja este cortical ou esponjoso. No entanto, a integração depende de *poros* que permitam a invasão de osso recém-formado. Para a invasão de osteoblastos, são suficientes poros de 80 μm a 200 μm, mas, para permitir a invasão de capilares, são necessários poros de 300 μm a 500 μm, e esse processo só será bem-sucedido se os poros forem interconectados. 9 10 11 A porosidade também tem influência direta sobre as características mecânicas do biomaterial: quanto mais poroso, maior o módulo de elasticidade e menor a sua resistência mecânica 12 ( Figs. 1A–C ).

Duas grandezas são consideradas entre as *características mecânicas* do material: sua resistência, que é a força suportada pelo material antes da fratura, e sua elasticidade, que é a capacidade de o material se deformar temporariamente sob a aplicação de uma carga e retornar à sua forma original após a remoção dessa carga. A baixa resistência propicia o aparecimento de fraturas e a falência mecânica do implante. A discrepância entre as elasticidades do osso hospedeiro e do implante, por sua vez, provoca micromovimentos na interface osso–biomaterial, o que impede a osteointegração. 11 12

A capacidade de o organismo *absorver* o substituto é uma propriedade altamente desejável, uma vez que possibilita a substituição gradual do implante por tecido ósseo recém-formado. 2 13 Para manter a resistência mecânica, é essencial que a taxa de reabsorção do material seja compatível com a velocidade de formação do osso novo. Além disso, a taxa de reabsorção é influenciada pela porosidade do substituto ósseo, uma vez que esta determina a área de contato entre o material e o tecido biológico circundante. 2 14

A *apresentação* do substituto define a sua melhor aplicação. Cimentos moldáveis são mais adequados para o preenchimento de espaços disformes, ao passo que blocos estruturados são preferíveis em osteotomias. O *custo* e a quantidade de *material disponível* para o implante finalizam a lista de propriedades importantes dos substitutos ósseos. O acréscimo de hormônios de crescimento e a impressão tridimensional (3D) de metais como o tântalo aumentam o custo, ao passo que o cimento ósseo de polimetilmetacrilato (PMMA) continua sendo o mais utilizado na prática clínica, principalmente por seu baixo custo.

## Tipos de enxertos

O *enxerto autólogo* (também chamado de *autógeno* ou *autoenxerto* ), retirado do próprio paciente, é considerado o enxerto ideal, pois não oferece reação imunológica e apresenta características de osteoindução, osteocondução e osteogênese. 6 No entanto, a disponibilidade limitada, o potencial morbidade do local doador, a cirurgia adicional, o aumento do tempo de cirurgia, o aumento da perda sanguínea, dor crônica na região doadora, disestesia e infecções impõem limites para seu uso na prática clínica. 1 2

Os enxertos autólogos esponjosos são frequentemente obtidos do osso ilíaco, da tíbia, do olecrano e do calcâneo, que são pobres em osteoblastos e osteócitos, mas são ricos em células mesenquimais primitivas e fatores osteoindutores, o que facilita a revascularização e a incorporação ao sítio receptor. 6 Já os enxertos autólogos corticais são obtidos da fíbula e da costela. 1 Como o enxerto cortical apresenta uma quantidade limitada de células osteoprogenitoras, sua integração ocorre por meio de reabsorção mediada por osteoclastos, seguida da aposição de osso recém-formado sobre o arcabouço necrótico do osso transplantado. Esse processo é mais lento em comparação à integração do osso esponjoso. 6 O enxerto corticoesponjoso da crista ilíaca combina a facilidade de integração do osso esponjoso com a integridade estrutural do osso cortical. 15 Alguns enxertos corticais (da fíbula e da costela) e os corticoesponjosos (da crista ilíaca) podem ser transplantados na forma de retalhos ou enxertos vascularizados, o que requer microdissecção ou anastomose. A fresagem do canal medular associada à irrigação e aspiração ( *reamer-irrigator-aspirator* , RIA, em inglês) é um método para se obter enxerto autólogo em maior quantidade e com maiores níveis de fatores de crescimento e células-tronco. 16

As alternativas biológicas ao enxerto autólogo são os *enxertos alógenos* ( *homógenos* ou *aloenxertos* ), quando provêm de outro indivíduo da mesma espécie, e os *xenógenos* ( *xenoenxertos* ), quando *provém* de outra espécie.

O enxerto alógeno está disponível na forma de segmentos ósseos e de preparados ósseos. Os segmentos são armazenados em bancos de tecidos em diversos tamanhos e formatos (total, esponjoso, cortical), inclusive para reconstruções osteoarticulares. 17 Entre as limitações dos aloenxertos estão a menor capacidade de incorporação, o maior risco de falha, a possibilidade de ser imunogênico e de transmitir doenças, e o fato de requererem, para preparação e estocagem, métodos químicos, liofilização, congelação ou irradiação, que podem afetar de forma variada suas propriedades mecânicas. 1 6 O aloenxerto processado mais comum é a matriz óssea desmineralizada ( *demineralized bone matrix* , DBM, em inglês), 6 que preserva fatores de crescimento, mas é limitada por falta de resistência mecânica, sendo mais usada na odontologia. 18

Os enxertos xenógenos, derivados de ossos bovinos, suínos e equinos, são processados com o objetivo de remover componentes biológicos e preservar a hidroxiapatita (HA) e, eventualmente, a matriz de colágeno, mas sua aplicação na ortopedia apresenta restrições. 1 19 Extraídos de outras espécies, ainda existem a quitosana, da casca de camarões, usada com o intuito de estimular a regeneração óssea e a diferenciação celular, e a HA extraída de corais. 2

## Tipos de substitutos ósseos

Os substitutos ósseos podem ser agrupados tecnicamente em quatro grandes classes: as cerâmicas, os biovidros, os polímeros e os metais. 15 As *cerâmicas* são amplamente empregadas como substitutos ósseos devido à grande biocompatibilidade. A grande diversidade de apresentações cria certa confusão quanto à nomenclatura. O material mais comumente usado é o fosfato de cálcio, que pode ser utilizado na forma cristalina, tal como é encontrada nos animais, a HA, ou na forma amorfa, cujo exemplo mais comum é o beta-fosfato tricálcico ( *beta-tricalcium phosphate* , β-TCP, em inglês). A combinação de ambos é chamada *fosfato de cálcio bifásico* ( *biphasic calcium phosphate* , BCP, em inglês), e sua apresentação em forma de cimento é conhecida como *cimento de fosfato de cálcio* ( *calcium phosphate cement* , CPC, em inglês).

A HA, principal componente mineral de ossos e dentes, pode ser extraída da natureza ou produzida sinteticamente na forma de partículas ou nanopartículas; 18 apresenta baixa taxa de reabsorção e resistência mecânica limitada, mas boa osteocondutividade. Por isso, a HA é amplamente utilizada como revestimento de implantes e menos para preenchimento de defeitos ósseos. Notavelmente, sua forma nanocristalina demonstra melhor desempenho biológico, que favorece a adesão celular e a diferenciação osteogênica. 20

O β-TCP, caracterizado por sua estrutura romboédrica e relação cálcio/fosfato inferior à da HA, tem sua reabsorção acelerada (13–20 semanas). 13 Seus microporos interconectados permitem a invasão de células osteogênicas e a angiogênese, embora sua estabilidade mecânica limitada restrinja sua aplicação em condições de alta carga. 21 Apesar disso, o β-TCP é amplamente pesquisado e disponível comercialmente. 1

O BCP, composto por uma mistura de HA e β-TCP em proporções variáveis (40–60% de cada), oferece um equilíbrio entre suporte mecânico e reabsorção óssea fisiológica, e destaca-se como uma opção promissora para diversas aplicações clínicas. 1 22

**Fig. 2:**
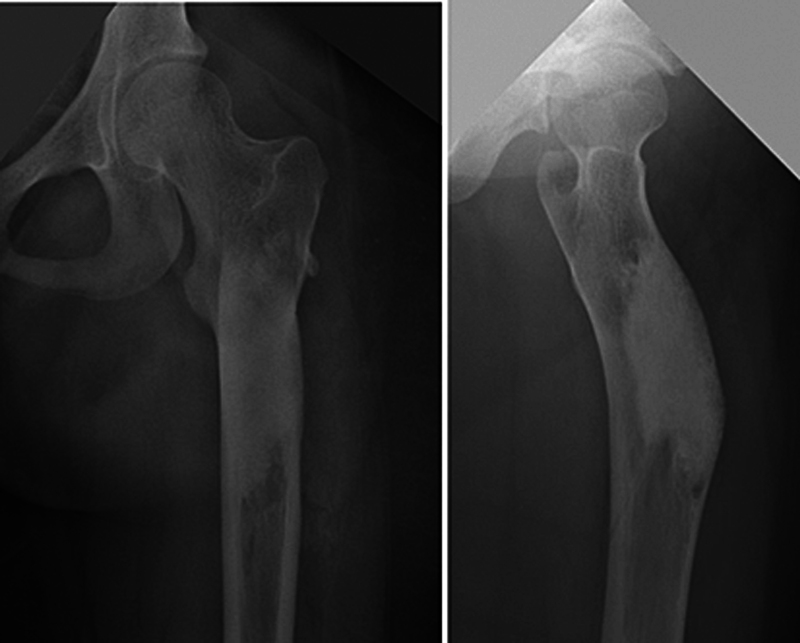
Radiografia do fêmur que demostra a integração de um cimento de fosfato de cálcio implantado há 2 anos.

Os CPCs são moldáveis durante o preenchimento de defeitos ósseos. 23 Formados pela mistura de componentes líquidos e em pó que se precipitam em nanocristais de HA, apresentam baixa resistência mecânica e porosidade, o que limita a reabsorção e a invasão por osso novo. São injetáveis e podem carrear componentes biologicamente ativos, como células osteoprogenitoras e fatores de crescimento. 23 Devido à sua maleabilidade, injetabilidade, bioatividade e biocompatibilidade, os CPCs são uma realidade no tratamento dos defeitos ósseos, e são altamente promissores para aplicações em engenharia de tecidos ( Fig. 2 ).

O sulfato de cálcio (forma pura de gesso) apresenta reabsorção completa em 6 a 8 semanas e fraco estímulo à formação óssea. Para contornar essas restrições, sua aplicação é frequentemente combinada com DBM, que melhora os resultados clínicos. 1

Os *biovidros* são cerâmicas com alta concentração de silicato (45–52%); caracterizam-se como vidros, e têm propriedade de bioatividade devido à sua capacidade de se ligar fortemente ao osso hospedeiro. 24 Acredita-se que sua bioatividade seja resultado da lixiviação dos íons de sílica para os fluidos corporais: 24 esses íons promovem a formação de uma camada de HA na superfície do biovidro, o que atrai células osteoprogenitoras e inicia a substituição gradual do material pelo osso recém-formado. A alta porosidade e a rápida absorção favorecem o aumento da taxa de substituição óssea, mas também induzem o aumento do pH local, o que pode inibir a remodelação óssea e tem impacto de longo prazo na eficácia. 25 Por serem frágeis e apresentarem baixa resistência mecânica, seu uso se torna limitado em áreas que requerem suporte de carga. 18

*Polímeros* , muitos deles também conhecidos como *resinas* , são macromoléculas formadas a partir da polimerização de moléculas menores. Os polímeros naturais mais comumente utilizados na engenharia de tecidos ósseos são o colágeno, a quitosana, a gelatina, a fibroína de seda, o alginato, a celulose e o amido. 26 Sua alta biocompatibilidade e baixa toxicidade os tornam atraentes para aplicações clínicas, e seus produtos de degradação são, em geral, não tóxicos e facilmente reabsorvidos pelo organismo. Um produto nacional que chegou a ser comercializado é a poliuretana de mamona, mas sua eficácia é tema de debate na literatura científica. 27

Os polímeros sintéticos biodegradáveis, como o ácido poliláctico ( *polylactic acid* , PLA, em inglês), o poli(ácido láctico-co-glicólico) ( *poly[lactic-co-glycolic acid]* , PLGA) e a policaprolactona (PCL), destacam-se na engenharia de tecidos devido à sua capacidade de ajuste em termos de propriedades físicas, incluindo porosidade, osteocondutividade, taxa de reabsorção e conformação final. 1 5 6 Essas características os tornam particularmente adequados para a produção de implantes personalizados, especialmente por meio de técnicas como a impressão 3D. 5 Além disso, a baixa imunogenicidade desses materiais é uma vantagem significativa, que contribui para a sua aceitação biológica. Contudo, durante o processo de degradação, pode ocorrer redução do pH local, o que pode comprometer a adesão de células osteoprogenitoras e as propriedades osteocondutivas do material. 18 O uso dos polímeros biodegradáveis ainda é relativamente recente, e há necessidade de mais estudos para consolidar as evidências e elaborar diretrizes de aplicação.

O PMMA é um polímero sintético não absorvível, amplamente utilizado na ortopedia para a fixação de próteses, 28 o preenchimento de defeitos ósseos, e o reforço mecânico em casos de ossos fragilizados, como em vertebroplastias, para aumentar a resistência mecânica de osteossínteses 4 ou para o carreamento de drogas como antibióticos e quimioterápicos. 29 30 Quando o PMMA é introduzido nos defeitos ósseos, forma uma massa sólida e compacta, que age como um espaçador biotolerado, não havendo nenhum tipo de integração óssea. 14 31

**Fig. 3:**
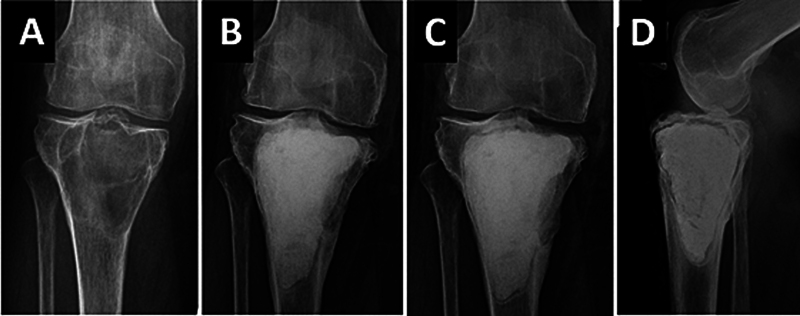
Radiografias da região proximal da tíbia que mostram um tumor de células gigantes comprometendo o osso subcondral do côndilo medial ( **A** ). Aos 2 ( **B** ) e 4 anos ( **C** , **D** ) após a cirurgia, observa-se reabsorção óssea em volta do bloco de cimento, mais acentuada na região subcondral. Essa reabsorção pode levar à fratura, à artrose e até à exposição intra-articular do cimento.

O cimento de PMMA apresenta vantagens como disponibilidade ilimitada, estabilidade mecânica imediata, facilidade de moldagem intraoperatória e custo acessível. Contudo, suas limitações incluem a reação exotérmica durante a sua formação, que pode causar superaquecimento e necrose óssea. 32 Embora se discuta seu possível efeito adjuvante no tratamento de tumores agressivos devido à alta temperatura, isso ainda não está comprovado. A soltura do cimento ósseo é uma limitação significativa, associada à reabsorção óssea e à formação de uma camada de fibrose, facilmente detectável em radiografias, que geralmente se estabiliza após 3 meses. 33 Uma causa bastante provável dessa condição é a diferença da elasticidade entre o cimento e o osso esponjoso, o que leva a micromovimentos e a reabsorção óssea. 31 Longe das articulações, a esclerose marginal que circunda o cimento não apresenta implicações. Entretanto, na região subcondral, a reabsorção óssea pode resultar em fraturas, incongruência articular e artrose. 29 Por fim, a presença definitiva do cimento impede a remodelação óssea e dificulta intervenções futuras, o que é a sua principal desvantagem 14 ( Fig. 3 ).

Os *metais* são bastante conhecidos pelos ortopedistas e demais profissionais que realizam osteossíntese. As placas, hastes e parafusos de aços à base de titânio (Ti) e cromo-cobalto (CoCr) são biocompatíveis e têm alta resistência mecânica e resistência ao desgaste. 4 O uso dos metais como substitutos ósseos tornou-se viável com o aparecimento da impressão 3D (manufatura aditiva, MA), o que possibilitou a confecção de peças no formato do defeito a partir de imagens de tomografias computadorizadas. Porém, o *stress shielding* , decorrente da diferença de elasticidade entre o metal e o osso, é a principal crítica ao uso dos metais como substitutos ósseos, pois provoca a transferência das tensões mecânicas do osso para a estrutura mais rígida, o metal, o que reduz o estímulo osteoindutor mecânico sobre o osso e promove a sua reabsorção. 4

Para o ajuste do módulo de elasticidade e para permitir a osteointegração, essas estruturas são produzidas com poros de tamanho e quantidade variáveis, sendo possível também mudar a formulação da liga metálica. O Ti é o metal mais comumente usado. O acréscimo do tântalo (Ta) às ligas de Ti conseguiu reduzir ainda mais o módulo de elasticidade. 34 Metais biodegradáveis à base de ferro (Fe) e magnésio (Mg), já passíveis de produção por impressão 3D, representam uma inovação promissora. Estruturas porosas de ferro demonstraram uma taxa de reabsorção de 5 a 16% em 4 semanas, e mantiveram um módulo de elasticidade comparável ao do osso esponjoso nesse período. Outra abordagem em desenvolvimento é a criação de ligas semiabsorvíveis de Ti e Mg, 35 o que evidencia um campo de pesquisa em rápida evolução e com avanços significativos.

## Osteoindutores

Muitos substitutos ósseos essencialmente osteocondutores podem ser utilizados em combinação com osteoindutores com o objetivo de aumentar a capacidade de osteointegração. A simples impregnação do substituto ósseo em aspirado de medula óssea pode ter efeito osteoindutor. 36 Os primeiros osteoindutores identificados foram as proteínas morfogenéticas ósseas ( *bone morphogenetic proteins* , BMPs, em inglês). 1 Trata-se de um grupo de moléculas classificadas como *fatores de crescimento* , pertencentes à superfamília dos fatores de crescimento transformadores β ( *transforming growth factor β* , TGF-β, em inglês). 6 As BMPs são produzidas por osteoblastos e desempenham um papel importante no recrutamento e na diferenciação de células osteoprogenitoras em locais de formação óssea. Apesar das diversas pesquisas, a eficácia desses componentes é controversa e carece de evidências clínicas. As principais limitações incluem a alta solubilidade – que dificulta a fixação da proteína ao local desejado –, o elevado custo e o risco de escape de BMP, com possibilidade de ossificação ectópica em áreas indesejadas.

Ainda entre os fatores de crescimento, o fator de crescimento de fibroblastos ( *fibroblast growth factor* , FGF, em inglês) já foi utilizado em estudos clínicos, apesar de não se conhecer bem o processo de osteoindução. Sabe-se que o FGF está presente durante todo o processo de consolidação de fraturas, mas seu efeito depende da dose e do momento de aplicação. 6 Apesar de estudos terem demonstrado seu efeito positivo, seu uso clínico ainda não foi liberado pelas agências regulatórias.

O fator de crescimento vascular endotelial ( *vascular endothelial growth factor* , VEGF, em inglês) tem duas funções relacionadas à osteoindução: efeito direto sobre a osteogênese e sobre a vascularização do calo ósseo em fraturas. 37 A preocupação em relação à indução de formação de hemangiomas e de recidiva de tumores, principalmente aqueles sensíveis ao VEGF, pode restringir o seu uso. 6

O hormônio da paratireoide ( *parathyroid hormone* , PTH, em inglês) está disponível comercialmente e é usado para ganho de massa óssea e redução de fraturas em pacientes com osteoporose. Estudos 6 mostraram que, com o seu uso, há ganho de massa óssea e diminuição no tempo de consolidação de fraturas; contudo, não há evidências de que o PTH inicie o processo de remodelação óssea ou de consolidação.

Íons bioinorgânicos, como os de silício, Mg, estrôncio, zinco e cobre, desempenham funções importantes no organismo, como cofatores de enzimas, coenzimas, sinalizadores de cascatas e atuação em canais iônicos. Algumas dessas funções estão relacionadas à osteogênese, e suas propriedades osteoindutoras têm sido amplamente estudadas e frequentemente confirmadas. Além disso, a incorporação desses íons em materiais-base apresenta vantagens, como baixo custo, maior prazo de validade e provável diminuição dos riscos em comparação com fatores de crescimento. No entanto, ainda faltam evidências robustas para que os substitutos que contêm esses íons sejam aprovados pelas agências regulatórias como osteoindutores. 6 38

Um fator osteoindutor que não deve ser menosprezado é o mecânico. A deformação óssea cíclica ativa os osteoblastos e promove a remodelação óssea.

## Ausência de preenchimento

**Fig. 4:**
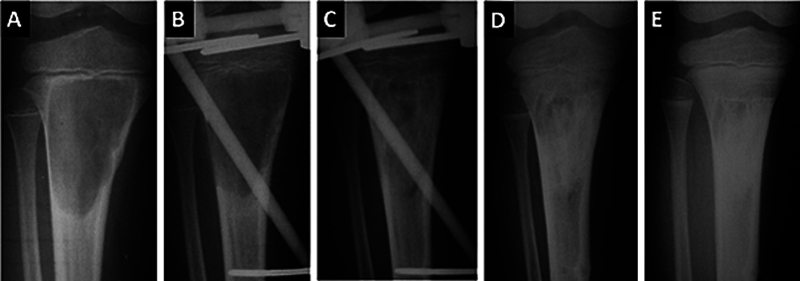
Radiografias seriadas de paciente de 5 anos com cisto ósseo aneurismático na tíbia, adjacente à cartilagem de crescimento ( **A** ). O paciente foi submetido a curetagem e a instalação de fixador externo transfisário, sem preenchimento do defeito ( **B** ). Cinco meses após a cirurgia, no momento da retirada do fixador ( **C** ), observa-se remodelação óssea progressiva, que se completou aos 6 ( **D** ) e 12 meses ( **E** ) de pós-operatório. A cartilagem de crescimento permaneceu íntegra.

Alguns autores 19 30 consideram o preenchimento dos defeitos ósseos desnecessário em determinadas situações clínicas, por identificarem altas taxas de neoformação óssea estimulada pelo efeito mecânico. Nesses casos, o osso remodelado tem aspecto diferente daquele do osso normal, mas pode ter resistência mecânica suficiente para suportar a carga funcional. O risco de fratura antes da remodelação óssea completa é mitigado pela osteossíntese profilática, que pode evitar complicações mecânicas e permite o apoio precoce de carga, situação nem sempre possível com o uso de substitutos ósseos. 19 Portanto, os enxertos e substitutos ósseos só podem ser considerado eficazes se tiverem potencial de remodelação maior do que o da remodelação óssea natural ( Fig. 4 ).

## Avanços

O surgimento de vários substitutos ósseos sintéticos oferece uma grande variedade de opções. No entanto, o resultado do tratamento ainda é incomparável ao do enxerto ósseo autólogo em termos da qualidade e do tempo da cicatrização óssea. De forma geral, os bons resultados dos substitutos disponíveis ainda dependem de um ambiente local favorável e do tamanho dos defeitos.

Os avanços recentes mais significativos em relação aos osteoindutores envolvem o aumento da produção e a redução de custo dos fatores de crescimento pela tecnologia da recombinação. As BMPs recombinantes humanas ( *recombinant human* *BMPs* , rhBMP, em inglês), que têm demonstrado razoável atividade osteoindutora, 39 e o entendimento do efeito osteoindutor dos íons bioinorgânicos são linhas de pesquisa importantes e promissoras. 6

O desenvolvimento de substitutos metálicos com propriedades mecânicas compatíveis com as do osso cortical, baixa toxicidade e poros grandes e interconectados, o que permite a osteointegração, tem mostrado bons resultados. Os metais absorvíveis que podem ser produzidos por impressão 3D 4 despertam grande interesse, assim como a bioimpressão 3D com células ativas, como osteoblastos e osteoclastos. 35

Com a aplicação da engenharia de tecidos à patologia musculoesquelética, foram descobertas potenciais estratégias de tratamento. A terapia genética regional envolve a implantação local de ácidos nucleicos ou células geneticamente modificadas para direcionar a expressão proteica específica, e tem se mostrado promissora como uma técnica de regeneração óssea. 40

Com os avanços recentes na engenharia de tecidos, surgiu uma nova abordagem centrada na “regeneração tecidual por tecidos naturais” em vez de na “substituição tecidual por biomateriais”. A cocultura e tricultura de diferentes tipos de células em estruturas de CPC oferecem grande potencial para promover a vascularização na regeneração óssea, especialmente no tratamento de defeitos ósseos de grande porte. 6 No entanto, mais estudos são necessários para validar essas abordagens e elucidar os mecanismos subjacentes, a fim de avançar na engenharia de tecidos e na medicina regenerativa.

## Conclusão

O tratamento dos defeitos ósseos é um grande desafio para os cirurgiões. O padrão-ouro ainda é o enxerto autólogo, mas seu uso esbarra em limitações como disponibilidade limitada e complicações cirúrgicas.

Apesar de todos os avanços tecnológicos nessa área, os substitutos ainda não alcançaram a eficácia clínica do enxerto autólogo. Seja por limitações de biodisponibilidade, osteoindução, osteocondução, ou até mesmo custo, a busca por um substituto ósseo que consiga conciliar todas essas variáveis é um desafio para a comunidade científica.

O elevado número de substituições ósseas realizadas em todo o mundo, aliado ao potencial comercial de um biomaterial eficaz, tem estimulado avanços significativos na área. Novas tecnologias relacionadas à biologia molecular, à MA, às terapias genéticas e à bioengenharia de tecidos fornecem um horizonte promissor para o desenvolvimento de novos substitutos ósseos.
